# Development of new malaria diagnostics: matching performance and need

**DOI:** 10.1186/s12936-016-1454-8

**Published:** 2016-08-11

**Authors:** David Bell, Alessandra E. Fleurent, Michael C. Hegg, John D. Boomgard, Caitlin C. McConnico

**Affiliations:** 1 Global Good Fund/Intellectual Ventures Laboratory, 3150 139th Ave SE, Bellevue, WA 98005 USA; 2 Concept Foundation, Geneva, Switzerland; 3 iSense, LLC, Cambridge, MA USA; 4 Independent Consultant, Seattle, USA; 5 International Training and Education Center for Health (I-TECH), Gaborone, Botswana

**Keywords:** Malaria, Rapid diagnostic testing, Target product profiles, Malaria diagnostics market

## Abstract

Despite advances in diagnostic technology, significant gaps remain in access to malaria diagnosis. Accurate diagnosis and misdiagnosis leads to unnecessary waste of resources, poor disease management, and contributes to a cycle of poverty in low-resourced communities. Despite much effort and investment, few new technologies have reached the field in the last 30 years aside from lateral flow assays. This suggests that much diagnostic development effort has been misdirected, and/or that there are fundamental blocks to introduction of new technologies. Malaria diagnosis is a difficult market; resources are broadly donor-dependent, health systems in endemic countries are frequently weak, and the epidemiology of malaria and priorities of malaria programmes and donors are evolving. Success in diagnostic development will require a good understanding of programme gaps, and the sustainability of markets to address them. Targeting assay development to such clearly defined market requirements will improve the outcomes of product development funding. Six market segments are identified: (1) case management in low-resourced countries, (2) parasite screening for low density infections in elimination programmes, (3) surveillance for evidence of continued transmission, (4) clinical research and therapeutic efficacy monitoring, (5) cross-checking for microscopy quality control, and (6) returned traveller markets distinguished primarily by resource availability. While each of these markets is potentially compelling from a public health standpoint, size and scale are highly variable and continue to evolve. Consequently, return on investment in research and development may be limited, highlighting the need for potentially significant donor involvement or the introduction of novel business models to overcome prohibitive economics. Given the rather specific applications, a well-defined set of stakeholders will need to be on board for the successful introduction and scaling of any new technology to these markets.

**Electronic supplementary material:**

The online version of this article (doi:10.1186/s12936-016-1454-8) contains supplementary material, which is available to authorized users.

## Background

Malaria seems ripe for the introduction of new and better diagnostic technologies. Despite being preventable, detectable and curable, it remains one of the main causes of mortality due to infectious disease. People at risk still fail to access early and accurate diagnosis, particularly in sub-Saharan Africa where the main burden of disease persists [1]. Misdiagnosis leads to unnecessary waste of resources, poor disease management, and contributes to a cycle of poverty in affected communities [2].

Despite advances in various aspects of diagnostic technology development, no new diagnostics have gained wide traction or a significant commercial market since the introduction of rapid diagnostic tests (RDTs) based on lateral flow formats in the early 1990s, which now dominate the point of care testing (POCT) market [3]. In spite of deficiencies in implementation, malaria diagnosis has improved dramatically over the last decade, albeit from a very low base, and RDT roll-out enabled the World Health Organization (WHO) recommendation in 2010 on universal parasite-based diagnosis. Reported diagnostic testing in the public sector in sub-Saharan Africa, for example, has increased from 36 % of suspected malaria cases tested in 2005 to 65 % tested in 2014 (after the WHO recommendations), with RDTs accounting for 71 % of diagnostic tests [4]. However, this also demonstrates that a considerable gap persists, impacting both malaria control and prospects for elimination.

Arguments for universal, parasite-based diagnosis are clear. Increasing use of lateral flow rapid diagnostic tests has provided poorly resourced, malaria endemic populations with access to diagnoses of similar accuracy to that available in hospitals in more developed health systems. This technology, when well used, is transforming fever management, has reformed understanding of malaria transmission, and has made malaria elimination look achievable [5]. Good malaria diagnosis also provides countries with the information necessary to target anti-malaria resources to communities that would most benefit and enables donors to measure the impact of funding. There is, therefore, a clear public health case for expanding and improving malaria diagnosis, either through current or new diagnostic platforms.

To understand how to address the remaining and wide diagnostic gap, it is necessary to understand where the gap is purely technological, and where it is the result of other factors, such as health systems financing, human resources and logistics, or simple disinterest. As funding for diagnostic product development is small in total [6], resources for product development must be directed to where there is real need and clear potential benefit. Research and development funding should have a good chance of achieving a useful public health outcome, and provide developers and manufacturers access to a sufficient market to justify their investment. This requires concentration on technologies that have characteristics that address identifiable and significant gaps, or offer significant advantages over the often low-cost, effective technologies already in use.

Priorities for malaria diagnostics have progressed significantly over recent years with renewed emphasis on tools required for malaria elimination. The transition of many countries traditionally considered to be high burden to a low-transmission state makes local or regional elimination potentially achievable [7]. Diagnostic requirements to address this situation have been divided elsewhere into two spheres:

- Tools to guide case management of acute fever [8, 9].
- Tools to guide and achieve elimination [8, 9].

While this dichotomy is broadly useful in guiding thinking on implementation, it encompasses a range of specific epidemiological contexts and programmatic needs and is insufficient to delineate the types of tests that are necessary to implement these quite different strategies. This paper categorizes diagnostic markets according to their role in overall malaria management, and the specific performance characteristics required to address them. The malaria diagnostic market is broken into six distinct markets, or use-cases, to which prospective technologies can be applied:

1. Case management (in low-resourced endemic countries).
2. Parasite screening (for low-density infections in elimination programmes).
3. Population screening (surveys for evidence of continued transmission).
4. Clinical research and therapeutic efficacy monitoring.
5. Microscopy quality control (cross-checking for light microscopy).
6. Non-endemic country (returned traveller) markets, distinguished primarily by resource availability.

**Fig. 1:**
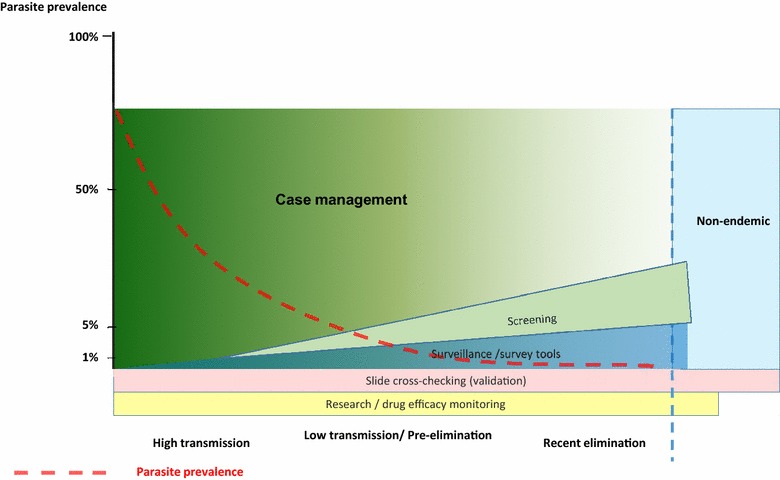
The importance of six malaria diagnostic markets in different epidemiological settings. As parasite prevalence declines (*left* to *right*), estimation of transmission becomes more dependent on low limit of detection screening and survey assays. Slide cross-checking and research markets cut across endemic and non-endemic settings

By segmenting the market into areas with clearly differing requirements and resources, a framework is developed for identifying diagnostic needs based on epidemiologic setting (Fig. 1) and minimum requirements for market entry. Such an approach could help to reduce the apparent redundancy seen in the current development of malaria diagnostics, and move the field forward more efficiently.

These variable requirements for malaria diagnostic tests in different market segments can be codified in target product profiles (TPPs) that specify the device requirements necessary to perform an identified task. Some performance parameters of TPPs are essential, some are desirable, and the importance of some will vary in different settings. The principles underlying the TPPs for the six markets suggested in this discussion are addressed in the following sections and summarized in Table 1. The detailed TPPs outlined in Additional file 1: Annex S1 are intended as examples of each identified market. They are derived from the MalERA TPPs developed in 2012–2013 [8], but adapted to the market segmentation suggested here. Other TPPs are being developed elsewhere and requirements change with time in response to evolving epidemiology and programme priorities.

**Table 1:** Examples of minimum target product profiles and market size

	Cost per processed sample (USD)	LLOD	Market size
Case management	(O): ≤$1.00 (M): ≤$1.00	(O) < 5 p/µL (elimination) (M) 100–200 p/µL	171 million slides examined via microscopy [10]
Parasite screening	(O): ≤$1.00 (M): ≤$5.00	(O) ≤ 2 p/µL^a^ (M) < 20 p/µL (LAMP, PCR achieve 1–2 p/µL)	~33 million people (within low-prevalence infection foci. Conservative estimate) [11]. Potential 1.5–8 million tests each year
Population screening	(O): ≤$1.00 (M): ≤$5.00	(O) ≤ 2 p/µL^a^ (M) < 20 p/µL (LAMP, PCR achieve 1–2 p/µL)	7000–10,000 cases surveyed [12]
Research/drug monitoring	Depends on application	(O) ≤ 5 p/µL (ex: drug trials)^a^ (M) ≤ 5 p/µL (ex: drug trials)^a^	Approximately 250 TES sites (~50/100 countries actually conducting TES). Numbers for wider research market unclear^b^
Microscopy quality control	(O): ≤$1.00 (M): ≤$1.00	(O): < 10 p/µL (M): 50 p/µL	29 million slides cross‐checked^c^
Non-endemic countries	(O): ≤$1.00 (M): ≤$5.00	(O) ≤ 2 p/µL^a^ (M) < 20 p/µL (LAMP, PCR achieve 1–2 p/µL)	30,000 suspected cases (screens) in USA. 200,000 suspected cases in Western Europe^d^

All values are dependent on program capacity and epidemiological setting

*O* optimum, *M* minimum

^a^Refer to Additional file 1: Annex S1 for exceptions and further details

^b^If we assume 4 sites per country, in around 50 endemic countries that actually run them. On top of that, around 50 research studies each year

^c^Based on unpublished estimates done by the authors using data from the World Malaria Report 2012 and 2013

^d^Based on unpublished estimates by the authors using data from Askling et al. [72]

## Detection of parasitaemia in endemic settings

### Case management in low resources endemic countries

#### Requirements

Case management requires assays that are supportable in a near-patient setting (typically POCT), minimize patient waiting time, and are low cost. The primary requirement for parasite detection is a sensitivity sufficient to detect symptomatic infections that are likely to have significant clinical consequences (morbidity or mortality), with a high reliability in confirmation of absence of malaria infection as a cause of illness (specificity). The capacity to detect parasite densities above a threshold of 100–200 parasites/µL appears sufficient in most cases of clinical *Plasmodium falciparum* infection [13]. However, the required lower limit of detection (LLOD) may be lower for diagnosis of clinical *Plasmodium vivax* infection [8, 9, 14, 15]. While identifying and treating infections with parasite densities below 100 parasites/µL is clearly desirable from an elimination point of view, i.e. reducing overall transmission [16], it could be argued that distinguishing infections likely to be responsible for symptoms from low density infections that are likely to be incidental to current illness holds some advantage in promoting the identification of other non-malarial causes of fever.

Case management diagnostic tools must provide sufficient immediate information to guide appropriate treatment. In situations where various Plasmodium species occur with different treatment requirements, tests must differentiate between species (principally *P. falciparum* and *P. vivax*). It is, however, difficult to quantify the accuracy required in species differentiation when developing TPPs. In case management, specific identification of *P. vivax* and *Plasmodium ovale* may be desirable for epidemiological purposes, but not necessary for managing the acute episode. In most areas of sub-Saharan Africa, mono-infections with non-*P. falciparum* species are relatively unusual and detection of *P. falciparum* alone can be sufficient [1], but where only non-falciparum species are endemic (e.g. areas of east Asia), it is still highly desirable to retain capacity to detect *P. falciparum* as severe cases may occur in non-immune returned travellers or their secondary cases. Therefore, capacity to detect *P. falciparum* is essential in most cases, while discrimination of other species in case management depends on species prevalence, co-infection patterns, and available resources.

#### Tools in current use

Endemic population case management is clearly the largest application of malaria diagnostic technologies, with the largest public health impact. While access to diagnosis is still low compared to potential need, an estimated 197 million microscopy tests were performed and at least 160 million RDTs distributed to National Malaria Control Programmes in 2014 (probably a large under-estimate as manufacturers report over 300 million sales) [4]. Light microscopy and RDTs, when of good quality, are adequate to detect most cases of clinically-significant malaria [13], and can be used successfully on a broad scale [17].

In use since the 1990s, lateral flow antigen-detecting assays (known as RDTs) can provide an accurate diagnosis for case management with a similar analytical LLOD to microscopy when in good condition and prepared and interpreted correctly, and are more amenable to remote locations and less dependent on high skill levels [18]. Currently, RDTs are available at well below US$0.50, are relatively stable in field conditions, provide some potential for species differentiation, and are adaptable to a village health worker level within the health system [19, 20]. RDTs can reach an LLOD sufficient for safe case management of *P. falciparum* [21–27], though sensitivity may still be insufficient for non-falciparum parasites [8, 9, 14, 15]. However, RDTs are not quantitative (which is of only limited importance in case management), species differentiation is limited, and quality control at point of use is difficult, placing the patient at risk due to manufacturing or storage failure [28].

While most RDTs have targeted histidine-rich protein-2 (HRP2) as a marker for *P. falciparum* infection, parasite populations have long been recognized with HRP2 gene deletions in the Amazon, undetectable by these tests [29]. Rising frequency of HRP2 gene deletions in African countries, recognized but unpublished at time of writing, raise the need for tests for non-HRP2 falciparum-specific targets (e.g. lactate dehydrogenase), though this will involve improving current technology rather than new platform introduction.

The persistence of light microscopy for malaria over a century after its introduction arises partly from its laboratory versatility across sample types and diseases, and from an absence of practical alternatives for applications, such as parasite quantitation and species differentiation [30]. While harder to support and less adaptable to community-level use, field microscopy has similar potential in terms of cost and throughput, and is useful for disease management beyond malaria. However, its dependence on technician performance can result in poor reproducibility [31–34], making the technology poorly suited to clinical decision-making in many settings where malaria is endemic. Its entrenched position, both in terms of microscopes in use and established commodity supply chains, means that it is likely to remain in wide use at some levels of the health system for the foreseeable future, despite the advent and increasing use of RDTs.

In view of the various deficiencies of current tests and the persisting large unreached at-risk population, significant unaddressed diagnostic gaps do persist that retard case management in regions where most cases occur [1]. There has been a continuing technological and programmatic failure in filling this gap, and an increase in funding and interest in product development over recent years has failed to produce successful replacement technologies. To be widely applicable in this market, a test will need to at least match the current performance of RDTs and good microscopy and equal them in terms of simplicity of use (RDTs) and cost. The failure of successful alternatives to emerge for malaria case management suggests that this is a technologically difficult barrier to overcome, and health system innovation may be at least as important a technology innovation to address this continued need.

### Parasite screening for low-density infections in elimination programmes

Progress towards elimination has highlighted the inadequacies of current field assays for specific applications such as screening and surveillance [35]. This is a relatively new market, and focal screen and treat (FSAT) strategies are not well established in most malaria endemic countries [36]. The place of screening relative to other strategies also remains the subject of debate. However, there is broad consensus that low-cost, highly-sensitive and specific screening tools would benefit malaria elimination in many countries currently endemic for malaria [8], whether as an adjunct to, or a driver of, elimination programmes.

**Fig. 2:**
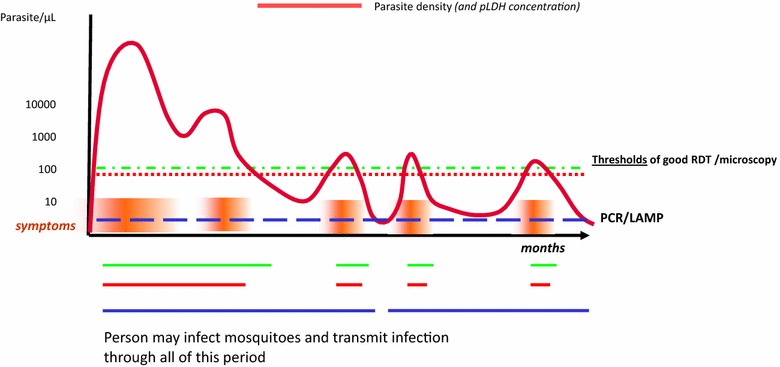
Stylized time course of an untreated *Plasmodium falciparum* infection in a host, and the impact of varying thresholds of detection on assay positivity. *Red bars* represent episodes of fever. Derived from observations of controlled infections to induce fever in tertiary syphilis patients, compiled by Collins and Jeffreys, US CDC. Unpublished data

The uses of diagnostic tests in elimination strategies, including their required performance and the strategies surrounding their use, are not well defined. Individuals with little previous exposure to parasites can maintain blood stage *P. falciparum* infection for up to 12 months or more, and be asymptomatic much of that time [37–43]. During the course of infection, parasite density will frequently be below the LLOD for expert microscopy [37, 38, 40, 44–46] and may be below the LLOD of routine polymerase chain reaction (PCR) leading to reservoirs of infection that remain undetected. Peaks in parasite density associated with fever could bring the patient to the attention of health services and aid diagnosis [37, 38, 47], but much of the infection course will remain hidden from routine case management diagnostic tools [16, 37, 38], leaving infectious individuals to promote transmission (Fig. 2) [48]. Light microscopy and RDTs may detect only a quarter of all cases. There is thus a need for low-cost highly sensitive field tests in low transmission settings targeting elimination [49]. Screening with these tools is, therefore, expected to be of limited use.

Leaving aside discussions on the relative impact of screening versus a combination of intensive vector management, case detection and mass drug administration (MDA), there is demand for near-patient screening tests capable of identifying asymptomatic cases with low-density infections in elimination programmes (e.g. FSAT programmes) [36, 46], and in border screening to protect transmission-free areas [50]. It seems likely that a mix of FSAT and MDA strategies will be used in elimination settings and tailored to the requirements of local epidemiology and resources.

To be widely applicable, such screening tests will need to have LLOD sufficiently low to detect a substantial and predictable proportion of asymptomatic infections. The LLOD necessary to achieve this is unclear and probably varies by epidemiological setting. A test with a LLOD of 1–2 parasites/µL can more than double detection rates compared with RDTs or microscopy [16]. As parasite density drops further, the probability of transmission from these infections will be smaller [16, 51], though this varies with time [38]. The importance of these infections to transmission also varies with the factors responsible for reducing local transmission in the first place, be they bed net use, access to case management, human behaviour, house construction, vector habits, or environmental change. MalERA recommended an LLOD of ≤5 parasite/µL as an optimum [8]. Detection of less than 2 parasite/µL, similar to nested PCR, is achievable now with loop-mediated isothermal amplification (LAMP) in near-patient settings [52].

An effective screening tool in an elimination setting will need to provide a result rapid enough to enable rapid treatment of parasite positive cases. The assay will need to distinguish between species in most geographical locations to provide information to guide treatment. Near-patient operability is desirable, though not essential if the screening test is used for broad population surveys to determine foci of transmission. Rapid results are of high importance to FSAT strategies to reduce both the likelihood that patient contact will be lost prior to treatment, and the likelihood of further onward transmission before treatment (elimination of *P. vivax* and *P. ovale* liver stage). A low false-positive rate is important, as assay results are likely to be used to determine parasite prevalence and not just guide directed treatment. As parasite prevalence in asymptomatic populations with relatively stable low transmission will be low, high specificity is difficult to achieve. However, overtreatment in this context is not directly harmful, assuming anti-malarials with few side effects are used.

### Surveillance for evidence of continued transmission

Malaria programs are required to identify areas with a high probability of ongoing transmission and areas where it is highly probable that transmission has ceased. Such evidence informs decisions on deployment of resources at the national and community levels. There is likely to be an increasing demand for surveillance, or population screening, as elimination programs progress and transmission becomes increasingly heterogeneous, requiring data to guide resource allocation and FSAT or MDA interventions and to aid confirmation of elimination. The market for population screening tools is characterized by a requirement to determine the probability of ongoing transmission and the lack of requirement for immediate treatment (desirable, but not clinically imperative in asymptomatic cases).

Two methods may be used to establish a high probability of continuing transmission: identification of current infection in members of a population, or identification of evidence of recent infection. While detection of parasitaemia is concrete evidence that prevalence is above zero, asymptomatic low-density parasite carriage can occur at very low prevalence in the population [40, 46]. Detection requires tools with very low LLOD and sampling of a very high proportion of the population in order to detect the few current cases. Sampling for a persistent marker of recent infection reduces specificity (in terms of current infection) but samples a wider temporal window and so should provide greater sensitivity with a lower sample size. The immediate candidate biomarkers are host antibodies specific to the malaria parasite [53–56]. Ideally, such antibodies would have relatively short half-lives, and so indicate infections within a defined time period. Screening based on longer-lasting antibody responses must be restricted to children of an age group consistent with the timeframe to be sampled [53, 57], and such groups may have atypical exposure, complicating interpretation of results.

Serological assays for population screening will need to be sufficiently low cost to enable a statistically significant population size to be sampled. Savings in better targeting of anti-malarial resources could justify the cost. It is likely that such a test would be coupled with follow-up by intensive case management interventions, MDA or FSAT, with targeting guided by the population screening results. This market is currently poorly served by existing technologies, and population screening is rarely performed outside of pilot and research studies. The market is, therefore, unpredictable but could evolve if suitable assays were available.

### Clinical research and therapeutic efficacy monitoring

Assays used as reference standards in monitoring of drug therapy must provide a result that distinguishes between all species, enables quantitation and monitoring of parasite density changes, has high consistency, and has a LLOD sufficiently low to enable tracking of parasite density below that associated with symptoms. However, these assays need not be capable of returning a result in time for immediate treatment if case management diagnostics (e.g. RDTs) are also available. This market segment is more difficult to define but is distinguishable from the needs of control or elimination programme implementation. Required LLODs depend on the aims of the research, but requirements for quantitative tests to address the needs of WHO recommendations for therapeutic efficacy monitoring sites are well-defined [58]. This market segment is limited in scale, but of significant public health importance if the development of drug resistance is to be monitored routinely by national malaria programmes, and drug policy is to be sufficiently responsive to early changes in patterns of efficacy. More broadly, standardization of tests used in clinical research would improve the comparability of malaria trials across space and time—currently poor due to lack of adherence to common standards [59].

### Cross-checking for microscopy quality control

Case management diagnosis in endemic settings continues to rely extensively upon light microscopy, with reported microscopy use increasing to 197 million microscopic examinations in 2013 [1]. To be reliable, light microscopy requires high levels of quality assurance, including slide cross-checking (validation) to monitor microscopist performance post hoc. Cross-checking is frequently poorly performed or absent due to limitations of technician skill, training, and time to perform the validation [60]. A potential market exists for an automated cross-checking platform that could process large numbers of Romanowski-stained light microscopy slides (Giemsa, Field’s or JSB Stain) at a central location and provide results that are highly consistent and of similar LLOD to expert microscopy. If such a device was sufficiently portable and cheap, it might replace manual malaria microscopy and change the use-case to include case management, but would still require some form of on-going validation.

An assay to validate microscopy technician performance must be capable of quantitation and species differentiation, and operate on slides that have been read previously and are not in current practice routinely cover-slipped. While the TPP of such an assay may overlap with that for case management or other tools, the requirement to be near point-of-care is removed. Automated slide feed would have clear advantages.

As with the market for a quantitative test for drug monitoring, the market size for such a test is limited, but the absence of a tool that fits the requirement of the market is a significant current impediment to ensuring the quality of malaria microscopy [60, 61]. The WHO recommends a minimum sample size for cross-checking of 10 randomly selected slides per month for a clinical laboratory; five slides reported as low density, and five reported as negative [30]. This could presumably be increased if an automated validation system were available. Should a digital automated slide reader of sufficient quality be developed, it will still be dependent on systems being in place for feedback, supervision and retraining. Estimating the true market for such a system is therefore somewhat difficult, and will depend also on the persistence of microscopy as a major platform for malaria diagnosis.

### Non-endemic country markets distinguished primarily by resource availability

The diagnostic market in non-endemic settings is essentially case management for febrile returned travelers. It is highly diverse and generally better-resourced than other market segments considered. Testing is likely to be two-tiered: there is a requirement for rapid testing at point of care to guide immediate management, and for tests that provide high sensitivity at low parasite densities, on the basis that patients are non-immune and may present clinically early in an infection course. Such requirements may be addressed initially by a rapid near-patient test, with confirmation by a test with high sensitivity (e.g. PCR), or by a highly sensitive near-patient test, such as LAMP [62], as resource constraints are less of an issue. The use of highly sensitive tests may also be driven by other imperatives such as litigation and public expectations, and by lack of confidence in point of care tests performed by technicians with very limited malaria experience. Malaria infections in developed countries are also generally more closely monitored, with more emphasis on parasite quantitation [63, 64].

A special case exists where screening of immigrant populations or other travelers is desired to avoid re-introduction and resumption of transmission [50, 65]. This requires parasite screening tools as described earlier, with a high probability of detecting very low parasite density (asymptomatic) infections, returning results in a sufficiently timely manner to enable identification and treatment of positive cases and to minimize the probability of onward transmission while awaiting results. A further area of need includes screening tools for donated blood–blood bank screening is a complex area with requirements varying with endemicity, travel history, and availability of alternative sources, and is not addressed further here.

### Estimating market size

The diagnostic market segments and corresponding TPPs discussed above (examples available in Additional file 1: Annex S1) are in many respects parts of a continuum: the performance requirements for the various markets often overlap, and most current diagnostic platforms imperfectly fit any profile, but are relevant to more than one. Thus, determining the place of a new platform in malaria diagnosis requires value judgments on the importance of reaching some performance requirements while falling short of others. The relative importance of aspects of product performance also vary between national programs and epidemiological settings. Some programs are able to maintain high quality light microscopy for case management at a peripheral level [60, 61, 66], while some struggle to support microscopy of safe quality more centrally [31–34]. Some programmes may operate in areas where populations are clustered around well-serviced nodes, while in others malaria may be confined to remote areas and predominantly mobile populations [67].

Performance, cost, and ease-of-use requirements exist that determine the usefulness of a given diagnostic technology. Examples of benchmarks set by current products that determine market entry criteria can be seen in case management, screening and non-endemic markets. These are addressed in turn below. A paucity of accurate data and dependence of future markets on changes in strategy development and donor funding renders the size of malaria diagnostics markets difficult to estimate and predict. For example, at what level does a population very close to malaria elimination, in which malaria case management no longer a priority, cease to be a market for case-management diagnostics? And when will elimination screening be introduced at scale, if at all?

### Case management

The annual diagnostic need in case management of acute fever in populations at risk of malaria could be several times higher than the market suggested based on the 319 million RDTs sold by manufacturers and 197 million microscopic examinations performed [1]. The market is restricted by availability of financial resources, trained staff, and competing public health priorities; it will therefore vary with the costs of the assays used. However, access is still inadequate across many endemic countries, illustrating the room for growth in this market. Further, the definition of ‘suspected malaria’ is obviously fluid: as example, a few hundred million people in China are at theoretical risk of malaria but the level of suspicion there, and consequently the likelihood of testing, differs in comparison to a high-transmission country.

High prevalence countries commonly have nearly the entirety of their populations living in at-risk areas. These countries also tend to be characterized by relatively resource-poor health systems and often have limited data available on diagnostic practice, or on quality assurance programs for these tests. Microscopy and RDT use is mixed: Burkina Faso, for example, used 0.57 million microscopy slides and 4.02 million RDTs in 2011 [10]. Many febrile patients did not receive a diagnosis [68], indicating that the total size of the potential market is well above the current level of use. The mix of private and public sector diagnosis also varies, with private sector market data even harder to obtain. Private sector health access varies greatly between countries, but likely contributes little to overall diagnostic numbers, due to a low use of malaria diagnostics in this sector [69].

Medium-prevalence countries (as defined by WHO) occur predominantly in South and Southeast Asia, and are the regions most affected by malaria after Africa with 29 million reported cases in the 2011–2012 season [70]. The majority of these populations live in low transmission areas, while a small proportion live in high transmission areas. Overall, 2.1 billion people in South and Southeast Asia live in areas at theoretical risk of malaria infection, approximately 62 % of the region’s total population. Microscopy is most often used in these regions. India, with its high at-risk population, dominates globally in number of microscopy slides examined each year; over 120 million in 2013 [1]. However, with only 2 % of these being parasite-positive, the market is fragile, and a decision to restrict testing or transition to RDTs as the primary diagnostic could remove much of the global microscopy market. Continuation at current scale in the face of such low slide positive rates must be uncertain, although increased emphasis on elimination may serve to increase market size. Microscopy rates are relevant for introduction of technologies aimed at improving microscopy quality assurance, based on an assumption that microscopy persists for reasons not served by RDTs, such as parasite quantitation.

Non-endemic country markets are essentially based on case-management needs of travelers (and to some extent screening for blood donation). Again, reliable data is not readily available. While malaria positive rates may be collated, data on tests performed are less accessible. As an example, we can consider the case management market in the United Kingdom (UK) and Western Europe. Between 1987 and 2006, 39,300 cases of malaria were reported to the UK’s Malaria Reference Laboratory [71]. With some extrapolation, a general idea of market size can be gained. Data on actual cases suggests about 10,000 cases of malaria each year in all of Western Europe, with about 70 % of these concentrated in the UK, France, Germany and Italy [72]. Assuming the positivity rate of 5 % is broadly accurate, it can be assumed that approximately 200,000 tests are performed. Much will depend on the pool from which immigrants arise and the areas to which nationals travel. While overall numbers appear small, the value of the market in monetary terms is more significant, as far greater resources are available for individual health care.

### Market for parasite screening tests

The majority of the populations in low-prevalence countries (e.g. Cambodia, the Philippines, Swaziland) live in areas of low transmission or are malaria-free, and transmission is highly heterogeneous [67, 73, 74]. As an example, 28 of the 80 provinces were declared malaria-free in the Philippines in 2011 [75] but prevalence in febrile cases still reaches 49 % in some areas against a background national rate of 4 %. A mix of diagnostic and screening practices is therefore likely to be required, including measures to prevent or reduce reintroduction from transmission to non-transmission areas, and high surveillance levels in areas where relatively little transmission persists. However, the market size for each of these applications is dependent on the priorities of the national programme, local priorities, and funding support (e.g. elimination in low-transmission areas versus reducing mortality and morbidity in high-burden areas), and so will remain unpredictable.

The WHO lists 20 countries as being in the pre-elimination and elimination phases, and nine preventing reintroduction of malaria [4]. Despite these achievements, 3.3 billion people were at risk of contracting malaria and developing disease in 2014, with 1.2 billion being considered high-risk [1]. Targeted screening for asymptomatic infections is likely to have a potential role in accelerating elimination and reducing the probability of reintroduction and resurgent transmission in these populations. As FSAT strategies and screening of immigrant populations remain limited by a lack of appropriate tools, and policies on wide-scale interventions (e.g. MDA) versus focused activity are still debated, these estimates of market size need to be treated with caution. Much will depend on priorities of large funding bodies, the availability of appropriate technologies, and the outcomes of future studies on impact of screening for low-density infections on interruption of transmission.

Finally, a small but more predictable screening market exists in countries conducting malaria indicator surveys (MIS) or similar surveys of large populations, where highly sensitive tests may be required to determine realistic prevalence in areas where low-parasite density infections are common. Typical MIS surveys sample about 7000–10,000 cases (e.g. Zambia) [12], but occur at irregular intervals.

Lastly, non-endemic country markets are typically characterized by much higher available resources, and an immunologically-naive population. Screening is still commonly based on microscopy, either initially or as a backup to RDTs [63, 64]. Total cases tested are very low for population size, with approximately 38,000 cases tested out of a population of 317 million in the UK will be spread across a wide range of health care access points (figure derived from positivity rates in the UK, based on unpublished analysis conducted by the authors in malaria diagnostics market analysis and report). Therefore, while resources are relatively high, the investment required per case diagnosed will be very high if a diagnostic platform is to be readily available across primary care settings, so fixed and variable costs are still relevant to market acceptability.

## Conclusion

Diagnostic markets can thus be categorized according to their role in overall malaria management, and the specific performance characteristics required to address them. Such market segmentation is necessary to understand whether a new technology is likely to be useful in malaria diagnosis, and if so, what market size might be expected. This paper suggests six distinct markets, to which diagnostic platforms can be applied. These vary in how adequately they are served by current technologies. While each of these markets is potentially compelling from a public health standpoint, size and scale are highly variable and continue to evolve. Consequently, returns on investment in research and development are unpredictable, highlighting the need for potentially significant donor involvement or the introduction of novel business models to overcome prohibitive economics. These investments need to be targeted to areas with real technology gaps, and where sufficient interest exists to sustain manufacture and justify the targeting of relatively scanty development funding.

Furthermore, given the rather specific applications, a well-defined set of stakeholders will need to be on board for the successful introduction and scaling of any new technology to these markets. Though the potential impact is quite significant, potential innovators in this space need to be aware of these challenges, and thoroughly understand the implications of the associated target product profiles before proceeding. Limited funding for research and development needs to be directed to technologies that are likely to address real areas of technological need (distinguishing from unrelated programmatic need). Assessing these gaps requires a realistic view of the effectiveness and shortfalls of existing technologies, rather than reiteration of the burden of malaria and fever cases. Current technologies are often very effective, the gaps can lie elsewhere.

## Additional file

10.1186/s12936-016-1454-8 Example target product profiles for malaria diagnostic markets.

## Abbreviations

FSAT: focal screen and treat
LAMP: loop-mediated isothermal amplification
LLOD: lower limit of detection
MDA: Mass Drug Administration
MIS: malaria indicator surveys
PCR: polymerase chain reaction
POCT: point of care testing
RDT: routine diagnostics tests
TPP: target product profiles
WHO: World Health Organization
