# Supplementary material for: Development of new malaria diagnostics: matching performance and need
Source: Malar J. 2016 Aug 11;15:406. doi: 10.1186/s12936-016-1454-8 (PMC4981959; doi:10.1186/s12936-016-1454-8)
Supplement: Supplementary file 1 — 10.1186/s12936-016-1454-8 Example target product profiles for malaria diagnostic markets. [file 12936_2016_1454_MOESM1_ESM.docx]

**Annex S1. Example Target Product Profiles for Malaria Diagnostic Markets**

The following six target product profiles (TPPs) are intended as examples of performance profiles that address requirements of the six malaria diagnostic markets listed in the main article:

1. Case management in low-resourced, endemic countries
2. Parasite screening for low density infections for elimination
3. Population screening for evidence of continued transmission
4. Clinical research and therapeutic efficacy monitoring
5. Microscopy quality control (cross-checking if light microscopy)
6. Non-endemic country (returned traveler) markets

The TPPs are adapted from the examples published through the malERA initiative. ^[[1]](#footnote-1)^ The TPPs of products aimed primarily at parasite identification, fitting more closely the malERA examples, are included in Table A.1. This includes tools for the case management, parasite screening, and research and drug efficacy monitoring markets (1,2 and 4 above). Tables A.2, A.3 and A.4 then present further modifications to these profiles in order to address the remaining markets for population screening, microscopy quality control and market needs in non-endemic countries (2, 6 and 5 above).

TPPs need to be adapted to specific markets and needs, and can be subject to interpretations of epidemiological data, available resources and characteristics of intended users. The examples below are intended as just that; examples based on the authors’ interpretations of current needs in typical settings. To guide product development, it is important to develop TPPs based on wide input and expertise, and also to consider likely evolution of requirements over the years of product development and implementation.

Acknowledgement: TPP tables are based on a template developed by the Foundation for Innovative New Diagnostics (FIND), Geneva, Switzerland.

**Table A.1**. Comparative Target Product Profiles for the three primary endemic country malaria diagnostic markets requiring detection of current parasitemia. p/µL: parasites/microlitre; Pf: *P. falciparum;* Pv: *P. vivax;* Po: *P. ovale (both species);* Pm: *P. malariae;* Pk: *P. knowlesi*. (M) Minimum, (O) Optimum

| **Criteria** | | ***1. Analytical and diagnostic assay performance*** | | | |
| --- | --- | --- | --- | --- | --- |
|  |  | **Case Management Diagnostic** | | **Parasite screening** | **Research / drug monitoring** |
| 1.1. Lower limit of detection p/µL | | (M) 100 p/µL - 200 p/µL  (O) < 5 p/µL (elimination) | | (M) < 20 p/µL  (O) ≤ 2 p/µL^a^  (LAMP, PCR achieve 1-2 p/µL) | (O) ≤5 p/µL (ex: drug trials)  > 5 p/µL acceptable in certain situations if well-defined and consistent (e.g. cross-checking). Set threshold for response. |
| 1.2. Analytical specificity | | Negative all other pathogens, common blood disorders | | Negative all other pathogens, common blood disorders | (M) Distinguish from other pathogens,  (O) Identify common blood disorders |
| 1.3. Clinical sensitivity in endemic population^b^ | | (M) >95%  (O) ≥99% | | (M) >95%  (O) ≥99% | (M) >95% if used for case management  >90% acceptable for cross-checking (adjust threshold for intervention accordingly) |
| 1.4. Clinical specificity | | (M) >95%  (O) >99% | | (M) >99% surveillance  (M) >95% screening (in low transmission areas) | (M) >95% |
| 1.5.1.  Pf-predominant areas^d^ | | (M): Pf  (O): Pf, pan | | (M): Pf  (O): Pf, pan | (M): Pf/non-Pf  (O)^e:^ Pf, Pv, Po, Pm, Pk |
| 1.5.2.  Mixed Pf / non-Pf areas | | (M) Pf/pan  (O) Pf/Pv/Po/Pm | | (M) Pf/pan  (O) Pf/Pv/Po/Pm | (M): Pf/Pv/Po/Pm  (O) Pf/Pv/Po/Pm/Pk |
| 1.5.3. Strain/isolate specificity | | No | | (M): No  (O): Yes (elimination certification) | (O): Drug efficacy…For resistance markers |
| 1.6  Gametocyte detection | | No | | (M): No  (O): Yes | (M): no  (O): yes |
| 1.10. Hypnozoite detection | | No | | (M): No  (O): yes | Not applicable |
| 1.11. Inter-reader variability | | kappa >0.9 | | kappa >0.9 | Repeat slides, >0.9 repeat sample, and between machine  Need calibrator included with platform (will be required to address regulatory issues) |
| ***2. Assay workflow*** | | | | | |
| 2.1. Sample type | | | Finger-prick blood  Venous  Other non-invasive | Finger-prick blood  Venous  Other non-invasive | Finger prick,  Venous |
| 2.2. Sample volume | | | Finger-prick (i.e. ≤ 40µL) | (M): Venous small tube  (O): Finger-prick (i.e. ≤40µL) | Low, practical, finger-prick (i.e. ≤40µL) |
| 2.3. Invasiveness | | | (M): finger-prick  (O): Non-invasive | (M): finger-prick  (O): Non-invasive | M: Finger-prick |
| 2.4. Procedural steps | | | (simple) | (Med-tech managed) | (O): Self-staining films /sample (minimize human/reagent error)  For integration into current microscopy-based studies, must have ability to read standard film |
| 2.5. Ease of Use | | | Community: Simple, few steps  Clinic: within med tech ability | (M): Clinic: within med tech ability  (O): Few steps, CHW. | (M): Within med tech ability  (O): Few steps, CHW. |
| 2.6. Time-to-result | | | (M): ≤30 min  (O): ≤15 min | (M): ≤30 min  (O): ≤15 min | (O): 30 minutes but capable of batching  (M): Dependent on application. Should allow batching and delayed reading.  Cross-checking: - time will depend on automated feed |
| 2.7. Biosafety | | | (M): High blood safety with basic universal precautions | (M): High blood safety with basic universal precautions | (M): High blood safety with basic universal precautions |
| 2.8. Sample and reagent identification | | | (M): Manual  (O): Automated | (M): Manual  (O): Automated | Automated linkage to result sample ID |
| ***3. Assay design and manufacturing*** | | | | | |
| 3.1. Stability of sample | | | Generally 15-30 minutes before processing to result. | Generally 12-48 hours before processing to result. | (M): Stored samples (stable at least weeks pre- or post- sample preparation, or until image stored) |
| 3.2. Stability of processed samples^g^ | | | (M) Not necessary  (O) Stable for cross-checking | (M) Not necessary  (O) Stable for cross-checking | (O): Stable for archiving (re-stain possible) unless stores image (transmissible images)  (M): Need some back-up archived material |
| 3.3. Stability of kits (temperature)^h^ | | | (M): >35°C  (O): >45°C | (M): >35°C (Limited periods)  (O): >45°C (Limited periods) | (M): >35°C (Limited periods)  (O): >45°C (Limited periods) |
| 3.4. Stability of kits (moisture) | | | Moisture-proof packaging | Moisture-proof packaging | Moisture-proof packaging |
| 3.5. Shelf-life | | | (M): 2 yrs. from manufacture (18m in country) | (M): 1 year (6 m in country)  (O): 2 yrs. from manufacture (18m in country) | (M): ≥ 1 year  (O): >2 yrs. for surface shipping all uses |
| 3.6. Stability of reconstituted reagents | | | (O) 12+ hours ambient temperature where these required  (M) Consistent with workflow | (O) 12+ hours ambient temperature where these required  (M) Consistent with workflow | Stable at ambient room temperature sufficient for effective workflow (e.g. 35°C) |
| 3.7. Positive / Negative controls | | | Ideal | (M) Required  (O): Positive control near acceptable threshold of detection | (M): Positive near threshold + negative + species controls (run daily) |
| 3.8. Calibrators | | | Ideal | Ideal | (M): Required |
| 3.9. Result capturing and documentation | | | See 2.8 | See 2.8 | (O): Image storage, transmissible |
| 3.10. Software | | | Where applicable:  (M): Updateable.  (O): Remote access for checking, updating, calibration | Where applicable:  (M): Updateable.  (O): Remote access for checking, updating, calibration | (M): Updateable.  (O): Remote access for checking, updating, calibration |
| 3.11. Training and educational requirements | | | (O): Half-day of community-level health worker | (O): <1 week of pre-trained medical technician | (O): <1 week of pre-trained medical technician |
| 3.12. Labeling of containers | | | (M) Product/ indication/ batch/ expiry | (M) Product/ indication/ batch/ expiry | (M) Product/ indication/ batch/ expiry  (O) (M) Product/ indication/ batch/ manuf. date/ expiry/ study/ site /calibration |
| 3.13. Physical conditions of reagents^i^ | | | (O): Field stable, dry  (M): Field stable | (O): Field stable, dry  (M): Field stable | Field stable |
| 3.14. Handling instructions | | | (M) Local technician language, and diagrammatic  (O) Field-tested with local users | (M) Local technician language, and diagrammatic | (M) Local technician language, and diagrammatic |
| 3.15.1. Assay package^j^ | | | (O): Individual | To be determined | To be determined |
| 3.15.2. Kit contents | | | (O): All consumables enclosed  (M): All non-routine consumables | (O): All consumables enclosed  (M): All non-routine consumables | (O): All consumables enclosed  (M): All non-routine consumables |
| 3.15.3 Environmental record | | | (O): Bulk package displays temperature violations | (O): Bulk package displays temperature violations | (O): Bulk package displays temperature violations |
| 3.15.4. Reagent safety | | | (M): Non-toxic | (M): Non-toxic | (M): Non-toxic |
| 3.16. Cost per processed sample (USD) | | | (O): ≤$1.00 | (O): ≤$1.00  (M): ≤$5.00 | Depends on application |
| ***4. Instrument architecture*** | | | | | |
| 4.1. Architecture | | | (O): (community-level) No dependence on electricity, no moving parts, no software dependence for result delivery  This may vary in established clinics. | Portable to near-patient setting  (O): 8-hour life battery  (M): 110/230/240 V  (O): Routine clinic waste disposal  (M): No toxic waste  (O): Closed system after initial sample input.  (M): Single room processing without significant risk of contamination. | One person can carry. Modular  (O): 8-hour life battery  (M): 110/230/240 V  (O): Routine clinic waste disposal  (M): No toxic waste  (O): Closed system after initial sample input.  (M): Single room processing without significant risk of contamination.  Portability depends on application. Robust enough for road. |
| 4.2. Equipment size | | | Portable, one person can carry | Portable to near-patient setting /clinic | (O) As above. Portable, one person can carry.  (M) Portable by car to clinic-level facilities |
| 4.3. Energy source^k^ | | | (M): no external power source  (O): All provided with test | (O): all provided with test | Low / in-built voltage stabilizer  ? off laptop  Need higher for automated feed |
| 4.4. Waste disposal | | | Village-level waste disposal | Basic health-system waste disposal | Basic health-system waste disposal |
| 4.5. Room conditions for instrument work | | | Where applicable:  (M): Resistant to dust entry  (O): Dust-proof | (M): Resistant to dust entry  (O): Dust-proof | (M): Resistant to dust entry  (O): Dust-proof |
| 4.6. Instrument cost (USD) | | | Where applicable:  Benchmark is $1000 (light microscope) | Depends on application, throughput | Research / monitoring use: benchmark is ~$1000  (Light Microscope) |
| ***5. Instrument workflow*** | | | | | |
| 5.1. Throughput | (M): 5-10 samples/ hour | | | (O) 800 samples/ 8 hours  (M) 200 samples/ 8 hours | (M): 5-10 samples/ hour  (O): 20+ samples/ hour |
| 5.2. Number of samples per run | N/A | | | Dependent on population sampling process. Preferably wide range (e.g. 10 – 100) | (O): Capable of running batches.  Dependent on sampling process, |

a 1.1: The required level of detection is controversial for Focal Screen and Treat (FSAT) applications. It is clear in low transmission areas that many (a majority) of infections are below the threshold of good microscopy and RDTs (20-50 p/µL), and that detection with a threshold of 1-2 p/µL will more than double detection rates. In sub-Saharan Africa, there is some evidence that RDT-based FSAT is ineffective. There is strong evidence that infections below 20 parasite/µL (asexual or gametocyte) can infect mosquitoes, but that the probability of infection declines with reducing parasite (gametocyte density). There is evidence that infections occur with parasite density below 1 parasite/µL in high- and low-transmission populations, but the contribution of these infections to transmission is unclear. It is likely that infectivity varies with time, and the importance of an infection to transmission is highly dependent on the force of infection (vector density, host population protection, etc.). MalERA recommended a threshold of detection of ≤5 parasite/µL as an optimum. FIND suggests ≤2 parasite/µL in situations with significant vector density and efficiency (i.e. equivalent to nPCR threshold on a non-concentrated sample). This could also be written as 95% detection at 2 parasites/µL)

b 1.3., 1.4: Sensitivity and specificity depend on reference standard quality, so difficult to measure in malaria infection in the presence of very low density and in very low prevalence (specificity), as reference standards are imperfect. Sensitivity standards therefore apply to the population with infection at or above the detection thresholds in 1.1.1.

c 1.5: Species differentiation: It is difficult to quantify accuracy required in species differentiation. In case management, specific identification of Pv and Po is desirable, but not necessary for managing the acute episode. In areas of sub-Saharan Africa, mono-infections with non-Pf species are rare and detection of Pf alone can be sufficient for case management in almost all cases. It is assumed the market is limited in areas with endemic Pv only, and in such environments it is still highly desirable to retain capacity to detect Pf, as this is potentially severe and may occur in returned travelers or secondary infections through local vectors. Therefore, detection of Pf is essential, while discrimination of other species in case management depends on species prevalence and co-infection patterns, and on resources available. However, for research and screening, species differentiation is of high importance for most purposes, at least for differentiation between Pf, Pm and Pv/Po (Pv and Po are unusual as co-infections, tend to be in geographically-distinct regions, and have similar management).

d 1.5.1: Implies that non-Pf mono-species infections are rare

e 1.5.1: Pf/non-Pf discrimination is sufficient, if access to confirmatory testing with full species differentiation is available later.

f 2.8: Automated ID processing and linkage to result and medical information system (MIS) is generally desirable for case management, but of low practical importance to direct patient management. Automated linkage is essential for research settings where batching of samples is undertaken, and highly desirable for screening applications where later case identification, often based on GIS information, is necessary.

g 3.2: Importance of stability of sample after reading depends on ability of assay to store all necessary sample information, and requirement for later cross-checking of the live assay results for QC purposes.

h 3.3: Temperature stability must reflect likely long-term storage. Case-management tools for field use must be sufficiently stable for long-term storage in the field with minimal temperature control. Tools for research and screening are generally in the field for much shorter periods with a research / screening team, and need not be stored long-term in a near-patient site. However, stability long-term in ambient temperature improves utility and reduces storage and transport costs of both. Requirement for refrigeration and freezing greatly increases transport and storage costs.

i. 3.13: Reagent conditions vary with level of use. ‘Field stable’ implies stable for sufficient time to enable efficient workflow in use setting. See 1.3.4, 1.3.5, 1.3.6 for overall kit stability. ‘Field stable’ in a community setting implies stable at minimum 35°C in malaria-endemic areas and at least 45°C in many sub-tropical endemic areas with no temperature control in place.

j 3.15.1: Assay packaging depends on application and assay platform (rate of sample throughput, etc.) and needs to reflect efficient workflow requirements.

k 4.3: External energy source implies dependent on mains power. For all settings, battery-dependency and solar recharging, etc. may be acceptable.

**Table A.2.** Variation in target product profile for a population screening assay (refer to Parasite Screening, Table A.1)

| Requirements are as for ‘Parasite Screening’ in Table A.1 above with following exceptions: | |
| --- | --- |
| ***1. Analytical and diagnostic assay performance*** | |
| 1.1-1.4 | Detection of surrogates for recent infection (i.e. specific antibodies.)  Semi-quantitative detection |
| 1.6 | Gametocyte detection: Not necessary |
| ***2. Assay workflow*** | |
| 2.6 | Time to result: Is of low importance (as does not inform treatment) |
| 2.8 | Sample and reagent identification: Should be automated where possible |
| ***3. Assay design and manufacturing*** | |
| 3.1 | Stability of sample: Must be high. (M) Weeks, (O) Months/years unless near-patient testing feasible |
| 3.3 | Stability of kits: Sample collection kit must be stable for (O) weeks at 35°C, (M) days at 35°C |
| 3.6 | Stability of reconstituted reagents: Relevant only to laboratory conditions, of less importance. |
| 3.10 | Software: Provide quantitative read-out, sample ID, and ideally geo-location data. |
| 3.11 | Training and educational requirements:  Sample collection should be simple (community health worker level)  Sample processing is in central laboratory; basic tertiary-trained laboratory technician is appropriate |
| ***4. Instrument architecture*** | |
| Processing in a central laboratory is assumed. Requirements are thus similar to those for general laboratory assays and equipment in low-resource health programmes: Robust and dust-proof, with low maintenance and very low specialist medical engineering requirements, and limited requirement for high cost of perishable reagents. These will vary widely in different endemic countries according to available resources, skill sets, and sample sizes to be processed. | |
| ***5. Instrument workflow*** | |
| As with screening profile. | |

a Prevalence surveys (e.g. MIS) are difficult to interpret in low transmission, as foci of transmission may be missed and high sample sizes are required to ensure a representative sample. As transmission decreases, relative changes in prevalence of moderate to high density infection may occur between age groups in parallel to overall prevalence reduction, complicating interpretation. At very low prevalence, most infections may be below the threshold of microscopy and RDTs, and more sensitive methods will provide a more accurate picture. However, the value of this data is unclear and will vary with epidemiological settings. Thus, the market value of a diagnostic reaching such thresholds as a survey tool will vary in different settings.

**Table A.3.** Variation in target product profile for a ‘microscopy quality control’ assay (refer to Parasite Screening, Table A.1)

| Requirements are as for Research / drug monitoring (Table A.1 above) with following exceptions: | |
| --- | --- |
| ***1. Analytical and diagnostic assay performance*** | |
| 1.1 | Lower limit of detection: (M): 50 p/µL, (O): <10 p/µL  Detection limit of a device intended purely for cross-checking manual microscopy should ideally be equivalent or better to an expert light microscopist (10-15 parasites p/µL). Detection at 50 p/µL would be acceptable as long as it was consistent, and discrepant results on low (usually non-symptomatic) infections taken in to account. |
| 1.3 | Clinical sensitivity: (O) 99%, (M)>90%  Limited sensitivity to clinical infections is acceptable as long as the threshold of detection is consistent and discrepant results can be adjusted by parasite density. |
| 1.5.1 | (M): Pf/non-Pf. Discrimination to a level of Pf/non-Pf would be acceptable where non-Pf mono-infections are rare |
| ***2. Assay workflow*** | |
| 2.4 | Procedural steps: The device must handle standard glass microscopy slides, with and without cover-slips |
| 2.6 | Time to result: Not important, as long as batching and automated feed is possible |
| ***3. Assay design and manufacturing*** | |
| 3.1, 3.2 | Stability of sample: High stability of stained blood film is necessary, as with delayed manual cross-checking. This reduces the applicability of Field’s stain. |
| 3.16 | Cost per processed sample: Microscopy quality control programmes are normally poorly funded. Throughput costs of above USD $1.00 will reduce ability to justify use as replacement for human reader. |
| ***4. Instrument architecture*** | |
| 4.7 | Instrument cost: May be viable up to USD $20,000, if throughput costs are then low^a^ |
| ***5. Instrument workflow*** | |
| 5.2 | \|Number of samples per run: Capable of continuous operation for >100 samples (e.g. overnight) |

a 4.7: Based on recommendation of digital microscopy consultation in February 2013. It is assumed that funding for quality control is commonly available, and justifiable, to set up systems rather than to run them long-term. A capital investment that is demonstrated to reduce running costs (increase sustainability) and improve quality is likely to find a market.

**Table A.4.** Variations in target product profile for an assay for non-endemic country markets (refer to Parasite Screening, Table A.1). Clearly, requirements will vary widely according to resources available, level of health system, and assay requirement (case management, confirmation of diagnosis, screening, etc.).

| **Scenario** | | **Reference Table 3.1** |
| --- | --- | --- |
| Rapid case management | Point of care/ peripheral | Generally equivalent to Case Management Profile (1), with greatly increased cost threshold, increased time to result (e.g. 1 hour) and reduced assay stability requirements. |
|  | Reference centre | Equivalent to Screening Profile, without high-throughput requirements and with greatly increased possible cost threshold. |
| Confirmatory testing | Point of care/ peripheral | Equivalent to Screening Profile (3), without high-throughput requirements and with greatly increased possible cost threshold. |
| Screening of immigrant populations | Equivalent to Screening Profile, without high-throughput requirements and with greatly increased possible cost threshold. | |
| Other requirements | Likely to require national regulatory approval. | |

1. malERA (2011). "A research agenda for malaria eradication: diagnoses and diagnostics." PLoS Med **8**(1): e1000396. [↑](#footnote-ref-1)
